# Vicarious Emotions of Fear and Pain in Rodents

**DOI:** 10.1007/s42761-023-00198-x

**Published:** 2023-08-04

**Authors:** Christian Keysers, Valeria Gazzola

**Affiliations:** 1 Social Brain Lab, Netherlands Institute for Neuroscience, Royal Netherlands Academy of Art and Sciences, Meibergdreef 47, 1105 BA Amsterdam, The Netherlands; 2 Department of Psychology, University of Amsterdam, Amsterdam, The Netherlands

**Keywords:** Freezing, Prosociality, Rats, Mice

## Abstract

Affective empathy, the ability to share the emotions of others, is an important contributor to the richness of our emotional experiences. Here, we review evidence that rodents show signs of fear and pain when they witness the fear and pain of others. This emotional contagion creates a vicarious emotion in the witness that mirrors some level of detail of the emotion of the demonstrator, including its valence and the vicinity of threats, and depends on brain regions such as the cingulate, amygdala, and insula that are also at the core of human empathy. Although it remains impossible to directly know how witnessing the distress of others *feels* for rodents, and whether this feeling is similar to the empathy humans experience, the similarity in neural structures suggests some analogies in emotional experience across rodents and humans. These neural homologies also reveal that feeling distress while others are distressed must serve an evolutionary purpose strong enough to warrant its stability across ~ 100 millions of years. We propose that it does so by allowing observers to set in motion the very emotions that have evolved to prepare them to deal with threats — with the benefit of triggering them *socially*, by harnessing conspecifics as sentinels, before the witness *personally* faces that threat. Finally, we discuss evidence that rodents can engage in prosocial behaviors that may be motivated by vicarious distress or reward.

Our ability to place ourselves in the shoes of others and share their emotions^1^ and feelings^2^ is essential to the richness of our social lives. That dysfunctions of this ability are so debilitating (Henry et al., 2016) is a testimony to the importance of this function. Following classic studies suggesting that rats also react to the emotional state of other rats (Church, 1959; Greene, 1969; Lucke & Baton, 1980; Rice & Gainer, 1962), the past two decades have seen an acceleration of studies providing evidence that rodents, including mice and rats, also align their emotions to those of others around them. Most of what we know stems from paradigms in which rodents show signs of distress^3^ when witnessing the distress of others, and we will focus our review on these paradigms (see Michon et al., in prep for a related review regarding positive emotional states). We explore what we know about the content of this vicarious^4^ affect — how specifically it mirrors the state of the witnessed individual, the degree to which it serves selfish or other-regarding purposes, and whether it is an unconscious emotion or a consciously represented feeling. We show that vicarious emotions in rodents depend on neural structures that are similar to those associated with affective empathy^5^ for pain in humans and argue that the ability to anticipate and prepare for threats by mirroring the distress of others may generate the evolutionary advantage that accounts for its the evolutionary stability.

## Paradigms That Reveal Vicarious Fear and Pain Across Rodents

**Fig. 1:**
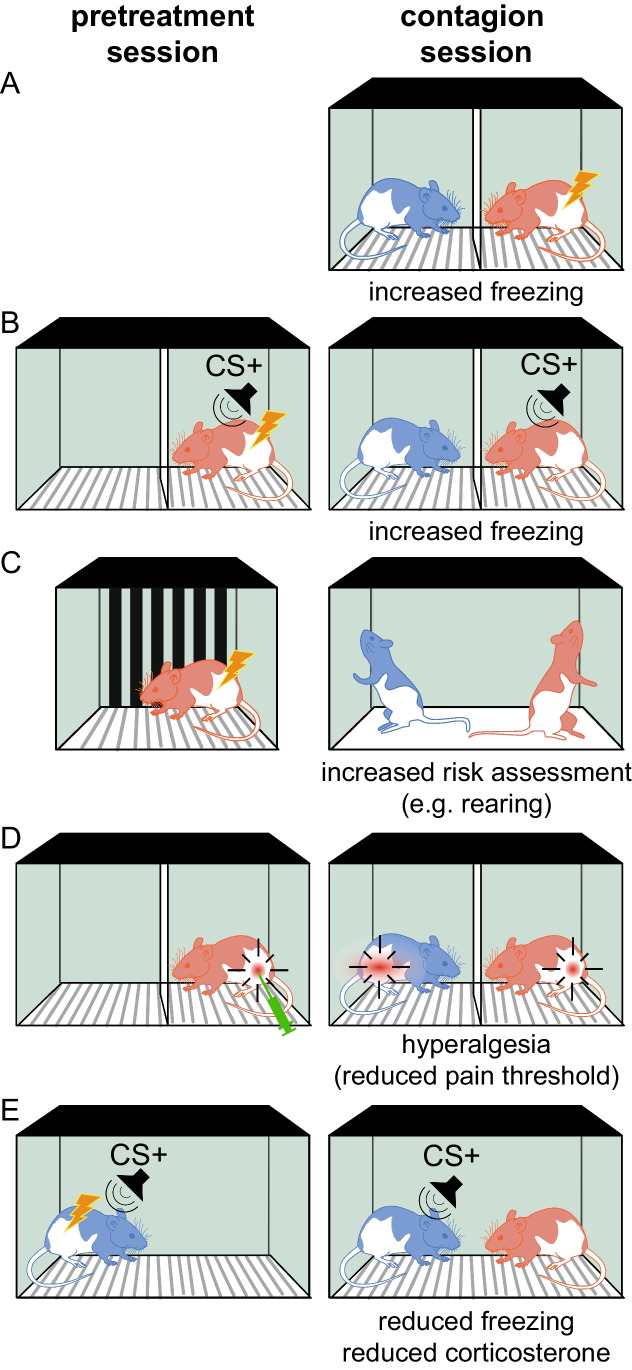
Paradigms revealing vicarious emotions. **A** An observer animal (blue) shows increases in freezing when witnessing a demonstrator (red) receive footshocks. **B** After a demonstrator has been threat conditioned by pairing a tone with footshocks (top), playing back the sound triggers freezing both in the threat-conditioned demonstrator (red) and in the non-conditioned observer (blue). **C** After a demonstrator has been stressed (top), an observer in contact with the previously stressed demonstrator shows signs of increased risk-assessment. **D** Injecting a pro-inflammatory substance in a demonstrator triggers hyperalgesia (a reduction in pain threshold) in both the injected demonstrator and in an uninjected by-standing observer. **E** After threat-conditioning (top), freezing and stress responses upon hearing the threat-conditioned tone are reduced when exposed to an unstressed conspecific. A review of an emerging literature suggesting emotional contagion of more positively valanced states can be found in a related review in this journal (Michon et al., in prep)

Several paradigms show that when distress is triggered in one rodent, called the demonstrator, the behavior of a by-stander, called the observer, is found to change in ways that suggest a mirroring of such a distress. What differs across these paradigms is (a) what stimuli are used to distress the demonstrator, (b) what behavioral readout quantifies the matching of distress in the observer, and (c) the timing of the observer-demonstrator interaction (Fig. 1).

Several experiments in mice and rats have revealed that an observer freezes more when witnessing a demonstrator receive shocks (Andraka et al., 2021; Atsak et al., 2011; Han et al., 2019, 2020; Jeon et al., 2010; Keum et al., 2018; Fig. 1A). Freezing is a defensive reaction that rodents display when under threat, particularly when escape is not an option, to reduce detection by predators, and is the behavior most often used to quantify fear in rodents. *Vicarious* freezing, i.e., the freezing in the observer, is interpreted as evidence that the distress of the shocked demonstrator triggered fear in the observer. This transfer of distress is considered evidence for the existence of *emotional contagion* in rodents.

In this paradigm, the footshocks trigger several observable responses in the demonstrator: squeaks including the audible range and jumping during footshocks, ultrasonic calls and freezing between shocks (Atsak et al., 2011; Carrillo et al., 2015, 2019), and alarm pheromones (Kiyokawa, 2017). Each of these signals contributes to emotional contagion: the playback of prerecorded squeaks (Han et al., 2019; Packheiser et al., 2022) or ultrasonic vocalizations (Kim et al., 2010) can trigger vicarious freezing in listeners, replacing the transparent divider by an opaque version reduces vicarious freezing (Jeon et al., 2010), and stress pheromone aggravate fear responses (Kiyokawa, 2017). This multiplicity of communication channels ensures that emotional contagion can occur even when a particular modality is unavailable.

Rats’ ability to align their distress to that of others is also borne out from paradigms in which a demonstrator is threat-conditioned in a pretreatment session, out of sight of the observer, by pairing a tone (conditioned stimulus, CS +), with footshocks (Fig. 1B). Later, in the actual contagion session, the observer witnesses the reactions of the demonstrator to playbacks of this CS + (Cruz et al., 2020; Kim et al., 2010; Pereira et al., 2012, 2020). Unlike in the previous paradigm, in which the demonstrator expresses both pain (during the footshocks) and fear (between them), here the demonstrator only expresses fear while the observer is present. That this triggers freezing in the observer (Cruz et al., 2020; Kim et al., 2010; Pereira et al., 2012, 2020) supports that fear is transmitted from demonstrator to observer. Playing back the sound of a rat moving around, and interrupting this sound (as is the case when the demonstrator freezes), suffices to trigger freezing (Pereira et al., 2012).

Vicarious freezing is stronger when the demonstrators display more freezing (Han et al., 2019), and in turn, the demonstrator freezing is reduced when the observers display less freezing (Cruz et al., 2020; Han et al., 2019). This mutual dependency on the state of the other suggests that this phenomenon is best conceived as animals aligning their emotional states to one another and differentiates this emotional contagion from the simplest forms of social facilitation called audience effect, i.e., that certain behaviors are altered by the mere presence of a conspecific (Zajonc, 1965). Vicarious freezing is also increased in observers that have themselves experienced footshocks in the past (Atsak et al., 2011; Cruz et al., 2020; Han et al., 2019; Sanders et al., 2013). The exact mechanism through which prior experience potentiates vicarious freezing remains unclear, but Hebbian associations (Keysers & Gazzola, 2014) between the distress of receiving footshocks and witnessing one’s own reactions (squeaks and later freezing) may account for how, later, witnessing similar reactions by a demonstrator can trigger fear (Cruz et al., 2020).

In both paradigms, the demonstrator has reasons to suspect imminent footshocks, a situation triggering freezing (Fanselow, 1994). When a threat is more distant, rodents instead assess risks by scanning their environment and rearing (Andraka et al., 2021; Kondrakiewicz et al., 2019). A paradigm exploring the transfer of this more remote fear exposes a demonstrator to footshocks in one environment alone, and then allows an observer to interact with that demonstrator shortly thereafter, in a new environment (Fig. 1C). In this new environment, the threat becomes more distant for the demonstrator, and demonstrators increase their risk assessment rather than freezing. That their observers also increase risk assessment, including rearing, rather than freezing, reveals that the state transmitted from demonstrators to observers in emotional contagion involves some information about the imminence of danger (Andraka et al., 2021; Keysers & Gazzola, 2021; Kondrakiewicz et al., 2019).

In a separate set of paradigms, experimenters measured pain rather than fear-related behaviors in the observer. Injecting acetic acid into the peritoneum of an observer and measuring the number of writhes is a classic index of pain intensity. Mice writhe more, when paired with another mice in pain, demonstrating that pain can also be contagious (Langford et al., 2006; Lu et al., 2018). Injecting an inflammatory drug (e.g., complete Freud’s adjuvant) in the paw of a demonstrator (Fig. 1D), and measuring pain sensitivity in both the injected animal and a bystander reveals increased pain sensitivity in both animals (Li et al., 2018; Smith et al., 2021; Zaniboni et al., 2018), even if the bystander is simply in the same room (Smith et al., 2016).

In humans, women are slightly more empathic than men (Christov-Moore et al., 2014). Between rats, and between mice that are familiar with each other, the level of emotional contagion is similar across same-sex male and female dyads (Du et al., 2020; Han et al., 2019; Sanders et al., 2013). Only amongst unfamiliar mice do male show less emotional contagion (Langford et al., 2006; Pisansky et al., 2017), with the risk of inter-male fighting perhaps requiring aggressive behaviors that mask the emotional contagion (Keysers et al., 2022). Interesting individual differences have however also been observed across strains of mice (Keum et al., 2016), providing ways to identify mechanisms linking genes to empathy-related phenomena in rodents (Keum et al., 2018) that could help understand the heritability of individual differences in human empathy and its lack in callous unemotional traits (Moore et al., 2019; Warrier et al., 2018).

## Emotional Contagion as a Selfish Mechanism for Threat Detection

In humans, empathy is tightly linked to prosociality: Empathic individuals are meant to experience concern for the distress of others. Some argue empathy actually evolved to fulfil the need, for all mammals, to nurse and care for their pups (de Waal & Preston, 2017). The distress of the pup *should* thus trigger an aversive state in the parent to motivate this care. This other-regarding vicarious distress is then thought to generalize to other members of the same species along a gradient of kinship and familiarity (de Waal & Preston, 2017). That oxytocin, associated with maternity, increases emotional contagion (Pisansky et al., 2017; Zoratto et al., 2018), and that emotional contagion amongst mice is stronger for siblings (Pisansky et al., 2017; Zoratto et al., 2018), is compatible with this view.

For any animal, detecting threats early, and preparing to respond to them, is also essential. To await direct contact with threats however is dangerous. Hence, if an animal is unable to detect a threat directly, but a conspecific has, sensing and mirroring their fear is a safer way to increase one’s readiness to deal with that threat (Keysers & Gazzola, 2021; Keysers et al., 2022). By doing so, emotional contagion co-opts a series of physiological, neural, and behavioral mechanisms in this social context that have, as emotions, evolved to flexibly respond to such threats. Emotional contagion may thus no longer primarily have evolved for the benefit of others (the pups) but for a more selfish, and hence arguably evolutionarily even more robust, imperative to prepare individuals for dangers. Vicarious freezing or vicarious risk assessment (Fig. 1A–C) then serve to save one’s own skin, by mitigating threats that a conspecific seems to have already encountered. Vicarious hypersensitivity to pain (Fig. 1D) then serves as a physiological preparation to deal with pathogens or injury. A number of arguments speak to this self-serving perspective. First, simulations show that emotionally coupled organisms deploy defense behavior more appropriately than either member alone, demonstrating the potential selfish value of emotional contagion (Han et al., 2019). Second, optogenetically reactivating neurons that had been recruited by shock observation trigger defensive behavior even when the individual is *alone* (Andraka et al., 2021), suggesting that these behaviors are not primarily for the sake of the demonstrator, as such a demonstrator is absent. Third, if hiding or escaping is an option, witnessing the distress of others can trigger these alternative behaviors — if emotional contagion had evolved to benefit the demonstrator in distress, such escape would fail to achieve the goal (Andraka et al., 2021; Pisansky et al., 2017). Fourth, emotional contagion-like behavior can be observed in animals that do engage in offspring-behavior-dependent care^6^ including fruit flies (Ferreira & Moita, 2020) and zebra fish (Oliveira et al., 2017; Silva et al., 2019). Indeed, even trees show stress reactions when other trees are attacked (Baldwin et al., 2006). Fifth, emotions can be mirrored across different species in what is called eavesdropping: the alarm calls of one species often trigger other species to hide or flee (Magrath et al., 2015). Mirroring the fear of members of different species is unlikely to have evolved to motivate parental care towards other species. Sixth, although in mice, emotional contagion is sometimes difficult to observe across unfamiliar male mice because of inter-male conflict (Langford et al., 2011; M. L. Smith et al., 2016), significant emotional contagion occurs even across unfamiliar rats (Han et al., 2019; Knapska et al., 2010) and female mice (Jeon et al., 2010; Pisansky et al., 2017; Zhou et al., 2018), where other-regarding motives would be weak.

## Emotional Contagion vs Mimicry and Empathy

Does the emotional contagion that rodents experience *feel* anything like what we feel when we witness the distress of our fellow humans?

Evidence for vicarious emotions in rodents hinges on the observation that the *behavior* of the observer comes to resemble that of the demonstrator. Does that behavioral observation reflect a transfer of an emotion (fear or pain) or a simply of a behavior (freezing or writhing)? Copying observed behavior, also called mimicry, is known to lead to a tight temporal alignment of the observed and copied behavior. In contrast, although demonstrator that freeze more triggers more freezing in observers (Han et al., 2019), moments of high freezing do not directly align (Andraka et al., 2021), and the jumping of demonstrators following shocks is not copied by observers (Carrillo et al., 2019). Similarly, although demonstrators that writhe more trigger more writhing in observers, the timing of the writhing does not tightly synchronize (Langford et al., 2006). These observations are more in line with emotional contagion: higher levels of fear or pain in the demonstrator trigger higher levels of fear or pain in the observers. This mirroring of fear states is then expressed in similar levels of freezing or writhing overall, but at the slower temporal scale of emotions rather than at the tight temporal scale of actions. Additionally, witnessing a demonstrator freeze (Pisansky et al., 2017), or optogenetically reactivating neurons activated by witnessing another animal receive shocks (Andraka et al., 2021), does not always trigger the same freezing behavior in the observer: if the observer has an opportunity to hide or escape, they do so instead, as expected if fear rather than freezing has been transmitted (Andraka et al., 2021; Pisansky et al., 2017). This flexibility is arguably what emotions evolved for: as a complex neural and physiological state that serves to flexibly orchestrate and prioritize adaptive behavior (Adolphs & Andler, 2018; Adolphs et al., 2019). By evolving to mirror the state of others, animals thus coopt this flexible emotional state to orchestrate and prioritize adaptive behaviors to threats inferred from others. In a way, the vicarious emotional state triggered by witnessing the state of the demonstrator can thus be considered to be *isomorphic* to that of the demonstrator; i.e., they have a similar underlying functional structure, and can be considered corresponding emotions, despite potentially triggering non-identical behaviors (and neural substrates).

**Fig. 2:**
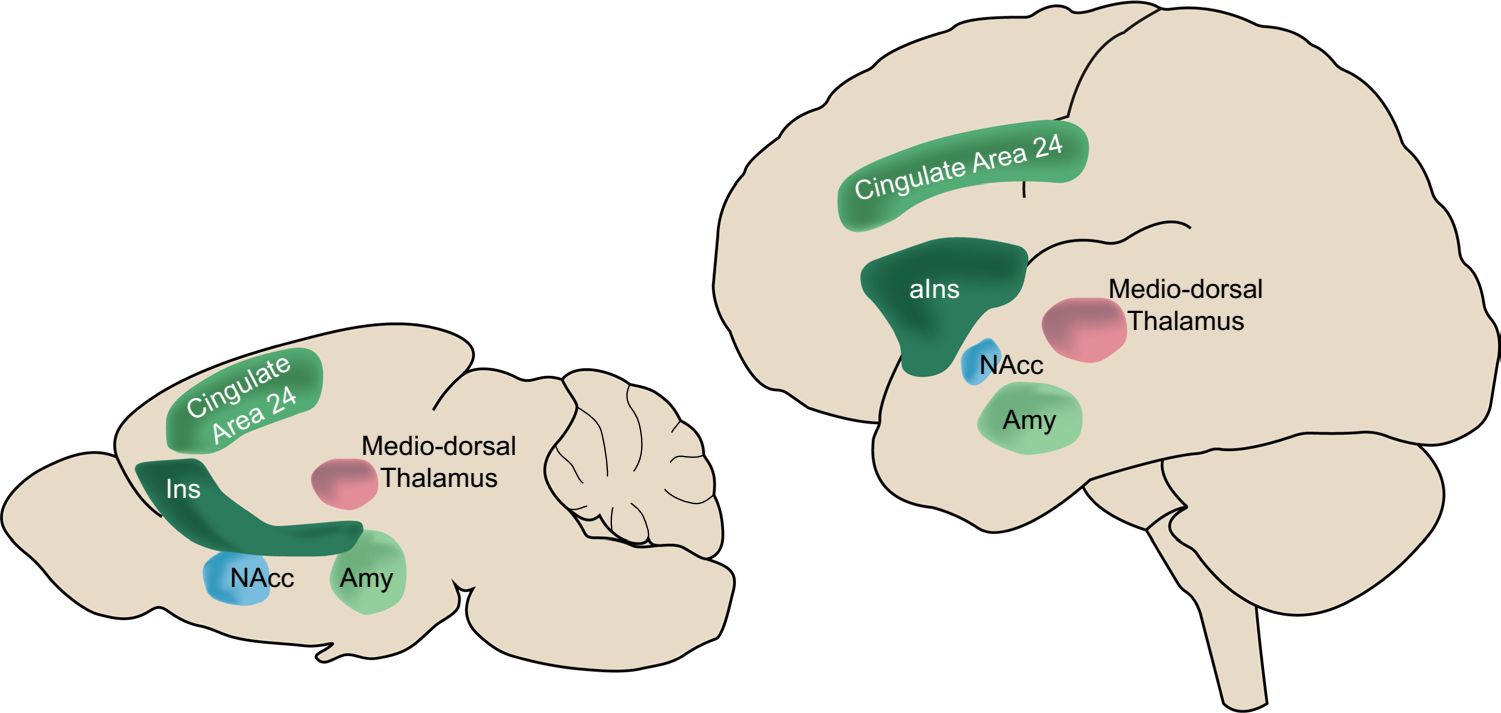
Similar brain structures associated with witnessing the distress of others across humans and rodents. Schematic representation of some of the core brain structures associated with witnessing the fear or pain of a conspecific in rodents (left, Keysers et al., 2022; Paradiso et al., 2021) and with empathy in humans (right, as in Jauniaux et al., 2019; Lamm et al., 2011; Paradiso et al., 2021). Abbreviations: Ins, insula; aIns, anterior insula; Nacc, nucleus accumbens; Amy, amygdalar complex. Note that in humans, activity associated with empathy clusters most reliably in the anterior part of the insula, whilst in rodents, we still lack this level of detail, and therefore mention the insula more generally

But do vicarious emotions for rodents *feel* anything like what empathy feels to us? This question can be approached through two angles (Kret et al., 2022). One may advocate that the simplest cognitive explanation for observed behavior should be privileged. Given that the rodent behavior presented here can be explained by the transfer of emotions without the need for conscious feelings, such cognitive parsimony would encourage us to doubt that rodents *feel* empathy which, unlike emotional contagion, requires *feeling* what others feel, and *knowing* that this feeling is on behalf of the other (Keysers et al., 2022). Interestingly, such parsimony is often applied to animal studies but seldom to our fellow humans, to whom we readily attribute such affective empathy, despite being unable to directly ascertain the nature of their feelings given the private nature of feelings. One may alternatively advocate that if two evolutionarily close species show similar behavior, it is parsimonious to suspect similarity in mental states proportional to the overall similarity of the species (Mill, 1972, p. 243). Based on such evolutionary parsimony, one may be more inclined to suspect that witnessing the distress of others may trigger feelings similar to our own, given the significant similarities in neural activity while witnessing the distress of others across rats, mice, and humans (Keysers et al., 2022; Paradiso et al., 2021; Fig. 2). In humans, meta-analyses of fMRI data reveal a network including cingulate area 24, the anterior insula, the mediodorsal thalamus, the amygdalar complex, and the nucleus accumbens to be reliably recruited while witnessing the pain of others, particularly in more empathic individuals (Jauniaux et al., 2019; Lamm et al., 2011). In rodents, all of these brain regions are also activated while witnessing the distress of others (see Keysers et al., 2022 for a review) and most are necessary for emotional contagion (see Paradiso et al., 2021 for a review). This similarity goes deep: the human literature suggests that reactivating neural representations of the observer’s own pain in the cingulate is central to empathy (Hutchison et al., 1999; Lamm et al., 2011; Singer et al., 2004). Rats and mice have a directly homologue cingulate area 24 (van Heukelum et al., 2020; Vogt, 2015), and inhibiting this region reduces emotional contagion in rats (Carrillo et al., 2019; Han et al., 2019) and mice (Jeon et al., 2010; Keum et al., 2018; S. Kim et al., 2012; Zhou et al., 2018), and in rats, area 24 neurons responding to the rat’s own pain are reactivated while witness a demonstrator receive shocks (Carrillo et al., 2019).

Although it is fundamentally difficult to know whether animals have feelings that resemble our own, based on this similarity of brain activity, when adopting a perspective of evolutionary parsimony, there is thus reason to suspect that some subjective components may also be similar. *Which* components of our experience may be shared with rodents will remain to be assessed. Whether the neural activity shared with humans suffices to generate conscious feelings or the awareness that the vicarious distress is of the demonstrator remains unclear. To be transparent about what we do not yet know, it is however prudent to remain agnostic and state that rodents show evidence for *emotional contagion*, while their ability for *empathy* remains to be studied.

## Emotional Contagion and Prosocial Behavior

Whether emotional contagion includes such other-regarding feelings relates to the question of whether vicarious emotions motivate actions that reduce the fear and pain of others. Here, we will call such actions prosocial, as they benefit others, independently of whether they are altruistically motivated or not. Paradigms in which rodents favor actions that reward others will be reviewed in a related review on the sharing of positive emotions (Michon et al., in prep).

Rats and mice have been shown to approach (Ferretti et al., 2019; Langford et al., 2010; Rogers-Carter et al., 2018; Scheggia et al., 2020) distressed conspecifics. In particular, if exposed to two conspecifics, only one of which is in an altered emotional state, they preferentially approach that conspecific, providing evidence that they indeed perceive *who* is in distress (Ferretti et al., 2019). These paradigms thus demonstrate the existence of an ability that those demonstrating emotional contagion (Fig. 1) did not: emotional contagion could exist without recognition of who was the source (e.g., when it is triggered by hearing pain squeaks from an unidentified source), but selective approach cannot.

Interestingly, rodents not only approach but also groom distressed conspecifics (Burkett et al., 2016; Du et al., 2020; Lee et al., 2021; Li et al., 2019; Lu et al., 2018; Luo et al., 2020; Matsumoto et al., 2021; Wu et al., 2021; Zeng et al., 2021). Both the vicinity and the grooming has been shown to reduce the distress of the recipient (Burkett et al., 2016; Kiyokawa & Hennessy, 2018; Kiyokawa & Takeuchi, 2017; Kiyokawa et al., 2018; Lee et al., 2021; Li et al., 2019; Sterley et al., 2018; Wu et al., 2021; Zeng et al., 2021) and has therefore often been considered a form of consolation akin to the consolatory embraces earlier reported in apes (de Waal & van Roosmalen, 1979).

When rats and mice are confronted with a demonstrator trapped in a small space or wet compartment, they can learn to liberate their conspecific in distress (Ben-Ami Bartal et al., 2011, 2014, 2021; Ueno et al., 2019; Yamagishi et al., 2020). When rats are given the choice between two actions that provide food, some will avoid actions that also harm others, even if this requires additional effort or delivers less food (Greene, 1969; Hernandez-Lallement et al., 2020). Interestingly, deactivating brain regions associated with emotional contagion, such as area 24 or the amygdala, also impairs these preferences for actions that prevent harm in others (Hernandez-Lallement et al., 2020), in line with the often-held notion that mirroring the distress of others can be a cause of prosocial motivation (Eisenberg et al., 2010; Smith, 1759). However, while emotional contagion is robust even between rats of different strains (Han et al., 2019), rats only appear to liberate rats of the strain they grew up with (Ben-Ami Bartal et al., 2021). This suggests that although the neural mechanisms of emotional contagion could promote prosocial behavior, the latter may be subject to additional constraints. Emotional contagion has the abovementioned selfish benefits independently of one’s relation to the demonstrator. Helping others, only has benefits if the recipient is likely to reciprocate or be genetically related and should thus be more finely regulated. The nucleus accumbens may be critical for this selective gating of emotional contagion into costly helping: in the accumbens, observing reward delivered to a demonstrator can trigger a transient dopamine release that resemble that when observers receive rewards themselves (but note that this release habituates quickly during observation, being significant only on the first trial, Kashtelyan et al., 2014), and the accumbens has higher activity in rats that liberate a same-strain conspecific than in those that leave an other-strain animal trapped (Ben-Ami Bartal et al., 2021).

In humans, helping is sometimes selfishly motivated by an urge to reduce the personal distress caused by witnessing the distress of others, rather than by a purely altruistic intention to benefit others (Batson et al., 1983). Although from a virtue point of view, this distinction is critical, from a consequentialist perspective, and hence arguably for evolution, this distinction is almost irrelevant^7^: the beneficiary’s benefits remain significant independently of the nature of the motivation. That rodents engage in these abovementioned behaviors, that objectively benefit others, could also be due to more selfish motivations. In particular, approaching conspecifics in distress provides access to valuable risk information conveyed through short-range pheromonal signals (Kiyokawa, 2017; Lee et al., 2021; Sterley et al., 2018). Liberating a trapped conspecific may provide social contact that is known to be rewarding to rodents (Solié et al., 2021). Avoiding actions that harm others may reduce the personal distress that emotional contagion would otherwise trigger (Hernandez-Lallement et al., 2020). From an evolutionary perspective, these selfish motives add a further force towards the evolution of prosociality, which ultimately also help other group members, and would thus coalesce with the forces of group-selection to stabilize such behavioral tendencies. However, to understand what emotions rodents experience, a better understanding of this distinction would be informative. Currently, it would be too early to conclude that rodents experience the altruistic sentiments we called empathic concern and sympathy in humans.

## Concluding remarks

We now have compelling evidence that a rodent’s emotional state comes to resemble that of those around them via neural mechanisms that are homologous to those associated with human empathy. That mirroring the emotions of others can serve to prepare for threats provides an evolutionary advantage that may explain why emotional contagion has evolved, and why its neural mechanisms are so preserved across rodents and humans. Rodents also seem to represent who is in altered emotional states and sometimes even help other conspecifics in distress. Rodents thus have more complex, socially relevant, vicarious emotions than previously thought. The degree to which the subjective and conscious experience characterizing human empathy is already present in rodents remains to be explored. Also, the development of emotional contagion across the lifespan of a rodent, and how it compares to humans, has received little attention but may help identify different facets of this ability that may emerge at different ages. Although we focused here on vicarious emotions, in which rodents respond to a specific emotion in a conspecific by evoking a similar emotion, because of their relevance for empathy and its disfunction in psychiatric disorders, it might sometimes be more adaptive to respond with complementary rather than similar emotions: responding to the anger of a dominant conspecific staring at us with fear and appeasement might be more beneficial than with anger. A detailed study of such non-matching social emotions would undoubtedly shed further lights onto the richness of social emotions in rodents.

## Footnotes

1 We use the term emotions as in Keysers et al. (2022) to refer to “states characterized by complex neural and physiological responses to significant actual or suspected events that act as latent variables to promote fitness by organizing priorities and motivating behavior — whether these states are consciously perceived (i.e., feelings) or not.”.

2 Unlike “emotion,” “feeling” is used here to refer to *conscious* experiences of those emotions, arising from the integration of external and internal/bodily cues, the context in which they occur, and our schemas of the world.

3 We use the term distress to refer to negatively valenced emotional states encompassing fear and pain that motivate avoidance.

4 Vicarious is used to refer to states triggered in an observer that approximate the state the observer would be in if in the stead of the demonstrator.

5 We use the term affective empathy to refer to feeling what another person is feeling while at the same time being aware that the vicarious emotion originates in the other.

6 While parental care may refer to any behavior or evolutionary strategy that increase the evolutionary fitness of offspring, we specify *offspring-behavior dependent* parental care here to focus on the scenarios that are meant to lead to the evolutionary advantage of emotional contagion. An animal laying eggs in a safe location does display parental care, but this care is independent of the behavior of the offspring, and hence would not benefit from emotional contagion.

7 We specify “almost” irrelevant, as there are situations in which this difference in motivation influences the consequences. As explained in Batson and colleagues (1983), if an agent has an option to escape the situation in which they are exposed to the distress of a victim, agents motivated by the urge to help, will help, while those motivated by an urge to reduce their own distress, may decide to escape the distressing situation. This distinction could be experimentally leveraged in rodents too, to differentiate the underlying motivation.

## Additional Information

### Data Availability

Data sharing not applicable to this article as no datasets were generated or analyzed for this review paper.
